# Cyclo His‐Pro Attenuates Muscle Degeneration in Murine Myopathy Models

**DOI:** 10.1002/advs.202305927

**Published:** 2024-05-10

**Authors:** Alessia De Masi, Nadège Zanou, Keno Strotjohann, Dohyun Lee, Tanes I. Lima, Xiaoxu Li, Jongsu Jeon, Nicolas Place, Hoe‐Yune Jung, Johan Auwerx

**Affiliations:** ^1^ Laboratory of Integrative Systems Physiology Institute of Bioengineering École Polytechnique Fédérale de Lausanne Lausanne 1015 Switzerland; ^2^ Institute of Sport Sciences and Department of Biomedical Sciences Faculty of Biology‐Medicine University of Lausanne Lausanne 1015 Switzerland; ^3^ R&D Center NovMetaPharma Co., Ltd Pohang 37668 South Korea; ^4^ School of Interdisciplinary Bioscience and Bioengineering Pohang University of Science and Technology (POSTECH) Pohang 37673 South Korea

**Keywords:** cardiomyopathy, Duchenne muscular dystrophy, mitochondrial dysfunction, muscle fibrosis, sarcopenia

## Abstract

Among the inherited myopathies, a group of muscular disorders characterized by structural and metabolic impairments in skeletal muscle, Duchenne muscular dystrophy (DMD) stands out for its devastating progression. DMD pathogenesis is driven by the progressive degeneration of muscle fibers, resulting in inflammation and fibrosis that ultimately affect the overall muscle biomechanics. At the opposite end of the spectrum of muscle diseases, age‐related sarcopenia is a common condition that affects an increasing proportion of the elderly. Although characterized by different pathological mechanisms, DMD and sarcopenia share the development of progressive muscle weakness and tissue inflammation. Here, the therapeutic effects of Cyclo Histidine‐Proline (CHP) against DMD and sarcopenia are evaluated. In the *mdx* mouse model of DMD, it is shown that CHP restored muscle contractility and force production, accompanied by the reduction of fibrosis and inflammation in skeletal muscle. CHP furthermore prevented the development of cardiomyopathy and fibrosis in the diaphragm, the two leading causes of death for DMD patients. CHP also attenuated muscle atrophy and functional deterioration in a mouse model of age‐related sarcopenia. These findings from two different models of muscle dysfunction hence warrant further investigation into the effects of CHP on muscle pathologies in animal models and eventually in patients.

## Graphical abstract

This study evaluates the therapeutic effects of Cyclo His‐Pro (CHP) on Duchenne muscular dystrophy (DMD) and age‐related sarcopenia. In a DMD mouse model, CHP restored muscle function, and reduced fibrosis and inflammation, preventing cardiomyopathy and diaphragmatic fibrosis. CHP attenuated muscle atrophy and functional decline in an age‐related sarcopenia model, suggesting potential therapeutic benefits for different muscle pathologies. BioRender is used to draw the graphical abstract.

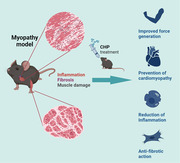

## 1 Introduction

Duchenne muscular dystrophy (DMD) is one of the most devastating inheritable muscular dystrophies that manifest in childhood.^[^ ^1^ ^]^ Affecting one in 3500 live male births, DMD is a recessive X‐linked genetic disease caused by mutations of dystrophin, a key member of the dystrophin‐associated protein complex (DAPC). The disassembly of the DAPC, and the consequent loss of a stable cytoskeleton‐extracellular matrix (ECM) connection, weakens the sarcolemma and makes muscle fibers susceptible to contraction damage.^[^ ^2^, ^3^ ^]^ The increased permeability of the sarcolemma disrupts many fundamental cellular signaling pathways, further promoting fiber degeneration and leading to the exhaustion of the regenerative potential of muscle stem cells over time.^[^ ^4^, ^5^, ^6^, ^7^, ^8^ ^]^ Not surprisingly, pro‐inflammatory pathways, such as the NF‐kB axis, and oxidative stress responses are activated in damaged DMD muscle fibers.^[^ ^9^, ^10^ ^]^ In the later stages of the disease, the loss of regenerative potential leads to fiber replacement with fat^[^ ^11^ ^]^ or connective tissue, boosting the development of fibrosis.^[^ ^12^ ^]^ The first clinical signs appear early in childhood, with functional impairments that first affect skeletal muscles and voluntary movements. Dystrophy eventually reaches respiratory muscles, a stage in which artificial ventilation is required.^[^ ^13^ ^]^ The cardiac muscle is also weakened, showing progressive accumulation of myocardial fibrosis and ultimately resulting in cardiomyopathy.^[^ ^14^, ^15^ ^]^ Indeed, respiratory and heart failure are the two leading causes of death for DMD.^[^ ^14^, ^15^, ^16^, ^17^ ^]^

There has been a collective effort to find therapies for DMD in the last decades. A few drugs exploiting exon‐skipping technology achieved the Food and Drug Administration (FDA) approval, but their therapeutic potential is limited to patients with specific mutations in the dystrophin gene.^[^ ^18^ ^]^ In 2023, the FDA approved the first gene therapy‐based drug under the name of Elevidys.^[^ ^19^ ^]^ Nonetheless, even if gene‐therapy approaches increase the life expectancy of DMD patients, combinatorial therapy with pharmacological agents is still necessary to delay DMD progression, manage DMD symptoms, and ameliorate patients’ quality of life. Today, the life expectancy of DMD patients is only 28 years.^[^ ^20^ ^]^ The current standard of care consists of corticosteroid treatment, to lower chronic inflammation and delay inflammation‐induced muscle damage.^[^ ^21^ ^]^ Notably, prolonged corticosteroid therapy comes with several side effects, including weight gain, growth delay, and osteoporosis.^[^ ^21^, ^22^ ^]^ Thus, research is now focusing on finding new strategies to reduce the activation of pathways involved in disease progression, precisely inflammation, fibrosis, and overall muscle damage.

Processes leading to the disruption of muscle homeostasis do not only occur in muscular dystrophies, such as DMD. In the last decades, the advancements in healthcare positively impacted human life expectancy, but now a growing proportion of the elderly is affected by age‐related sarcopenia, loss of muscle mass and strength, often leading to frailty.^[^ ^23^ ^]^ With aging, muscle tissue experiences major structural changes and a decline in strength and function.^[^ ^24^ ^]^ The development of sarcopenia is linked to many underlying factors (i.e., hormonal changes, physical activity), but also chronic low‐grade inflammation plays an important role in muscle metabolism.^[^ ^25^ ^]^ While the exact pathophysiological mechanism is still unclear, major changes also occur in the structure of skeletal muscle, including ECM remodeling and fibrosis.^[^ ^26^, ^27^ ^]^ Sarcopenia is still widely undiagnosed and diagnostic screening tools are inaccurate.^[^ ^28^ ^]^ Diagnosis is mainly based on muscle strength—assessed usually by grip test—and muscle mass.^[^ ^29^, ^30^, ^31^ ^]^ The first‐line treatment for sarcopenia focuses on resistance training, as no pharmacological intervention has been approved yet.^[^ ^32^, ^33^ ^]^ Extensive efforts were made toward developing myostatin inhibitors, showing promise to increase muscle mass and strength in mice^[^ ^34^ ^]^; however, these pre‐clinical results failed to translate to humans.^[^ ^35^ ^]^ Clinical trials sometimes reported an increase in muscle mass but not in functionality, possibly due to different regulatory mechanisms between mice and humans,^[^ ^36^, ^37^ ^]^ or to the relative importance of improving muscle function and metabolism over muscle mass. In line with this last premise, targeting age‐related mitochondrial dysfunction through the administration of mitophagy boosters such as Urolithin A, appeared as a promising strategy to preserve muscle functionality in aging.^[^ ^38^, ^39^, ^40^, ^41^ ^]^ Research is now active in finding new therapies that can effectively delay muscle atrophy and partially restore strength and function, without incurring major side effects.

The natural compound Cyclo Histidine‐Proline (CHP), initially discovered as a degradation by‐product of the thyrotropin‐releasing hormone, quickly sparked interest for its anti‐inflammatory and antioxidant properties in the field of neurodegeneration and neuroinflammation.^[^ ^42^, ^43^, ^44^ ^]^ In a recent study on steatohepatitis, we described the anti‐inflammatory and antifibrotic properties of CHP against liver disease.^[^ ^45^ ^]^ Remarkably, the transcriptomic signature of this effect was not restricted to the liver, as suggested by the conserved down‐regulation of inflammatory and fibrotic pathways in different tissues, including muscle.^[^ ^45^ ^]^ With its suitable safety record in humans,^[^ ^46^ ^]^ and the absence of known side effects (clinical trials NCT02784275, NCT03560271), CHP emerges as a promising candidate for drug repurposing. Building upon its antifibrotic, anti‐inflammatory, and antioxidant properties, we now present its efficacy in mitigating muscle weakness and mitochondrial dysfunction in mouse models of DMD and sarcopenia.

## 2 Result

### 2.1 CHP Preserves Muscle Function in a Mouse Model of DMD

**Figure 1:**
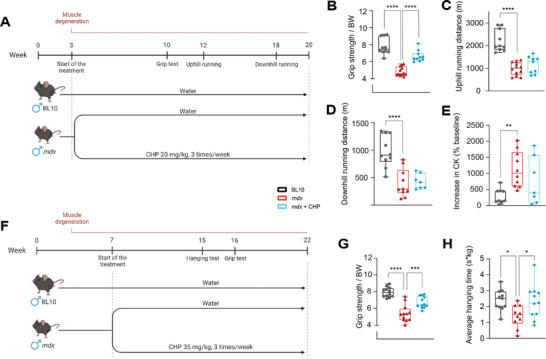
Treatment with CHP prevented muscle weakness but did not improve endurance in a DMD mouse model. A) Schematic representation of the animal experiment in a preventive setting. *mdx* mice were treated with CHP (20 mg kg^−1^) from week 3 to 20; *mdx* and BL10 mice were treated with vehicle as controls. Grip test, uphill running, and downhill running were performed as indicated. B) Grip strength normalized on body weight (BW), performed at 10 weeks of age. n = 10 for BL10, 12 for *mdx*, 10 for *mdx*+CHP. C) Total distance run in uphill (+5°) running exercise, performed at 12 weeks of age. *n* = 10 for BL10, 12 for *mdx*, 9 for *mdx*+CHP. D) Total distance run in downhill (─5°) running exercise, performed at 18 weeks of age. *n* = 10 for BL10, 10 for *mdx*, 7 for *mdx*+CHP. E) An increase in plasma CK levels after the downhill running was expressed as a percentage change in the pre‐run CK baseline. *n* = 9 for BL10, 10 for *mdx*, 7 for *mdx*+CHP. F) Schematic representation of the animal experiment in a therapeutic setting. *mdx* mice were treated with CHP (35 mg kg^−1^) from week 7 to 22; *mdx* and BL10 mice were treated with vehicle as controls. The hanging test and grip test were performed as indicated. G) Grip strength normalized on body weight (BW), performed at 16 weeks of age. *n* = 12 for all groups. H) Average hanging time on a 4‐limb hanging exercise, measured at week 15 and normalized on BW. Whiskers in boxplots represent the min to max range. *n* = 11 for BL10, 10 for *mdx*, 11 for *mdx*+CHP. One‐way ANOVA, followed by Dunnett's multiple comparison test versus *mdx* group, was used for statistical analysis (B‐E, G‐H). *p* values are indicated as follows: ^*^ *p *< 0.05; ^**^ *p *< 0.01; ^***^ *p *< 0.001; ^****^ *p *< 0.0001. A,F) Created with BioRender.com.

General muscle weakness and decreased force production are among the first clinical signs of DMD. Here we tested the efficacy of CHP in the *mdx* mouse, a widely used DMD model with a well‐characterized pathophysiology.^[^ ^47^, ^48^, ^49^, ^50^, ^51^, ^52^, ^53^, ^54^ ^]^ In the first exploratory study, the treatment started at 3 weeks of age, concurrently with the expected first burst of fiber degeneration.^[^ ^48^ ^]^ C57BL/10ScSn‐*Dmd^mdx^* (*mdx*) mice were treated with CHP at a dose of 20 mg kg^−1^, given by oral gavage three times a week for 17 weeks (**Figure** **1**A; Table S1, Supporting Information). C57BL/10ScSn (BL10; control) and *mdx* control mice were gavaged with vehicle (water) following the same administration protocol, as negative and positive controls, respectively (Figure 1A; Table S1, Supporting Information). Force production and exercise endurance were assessed with three different phenotyping protocols: grip strength was measured at week 10, and endurance was evaluated at weeks 12 and 18, with uphill and downhill running protocols, respectively. Physical fitness was affected in *mdx* mice, which showed an overall decrease in limb strength and exercise endurance, together with an increase in creatine kinase (CK)—a biomarker of sarcolemma damage^[^ ^55^ ^]^—upon eccentric exercise (Figure 1B–E). Mice treated with CHP exhibited enhanced grip strength, suggesting that the drug prevented the progressive development of muscle weakness typical of the *mdx* model (Figure 1B). However, we did not observe any improvement in endurance during physical exercises (Figure 1C,D), nor a significant decrease in CK levels (Figure 1E). Given the positive results on muscle weakness prevention, a second independent study was performed to evaluate the therapeutic potential of CHP (Figure 1F). In this setting, CHP was given from 7 weeks of age—after the first peak of fiber degeneration^[^ ^48^ ^]^—following the same administration protocol of the first study (per *os*, three times a week) but at an increased dose (35 mg kg^−1^) (Figure 1F; Table S2, Supporting Information). Muscle strength was evaluated at a later stage (15–16 weeks of age) through measurements of grip strength and maximal hanging time (Figure 1G,H). In both tests, mice treated with CHP showed improved force production, providing compelling evidence that CHP can restore limb strength in the DMD model, whether given as a preventive measure or in a therapeutic setting.

### 2.2 CHP Improves Ex Vivo Strength and Ca^2+^ Handling of Limb Muscles

**Figure 2:**
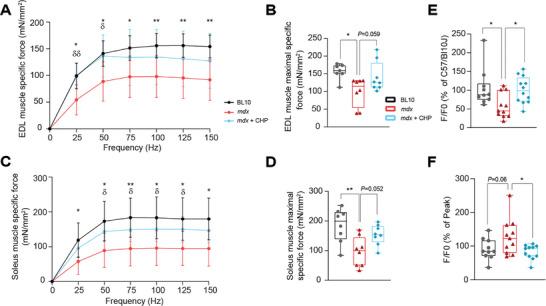
Ex vivo contractility and force production were restored by CHP, with improved Ca^2+^ handling. A) EDL muscle force‐frequency relationship. *n* = 7 for BL10, 8 for *mdx* and *mdx*+CHP. B) Maximal specific isometric force in EDL developed during the test. *n* = 7 for BL10, 8 for *mdx* and *mdx*+CHP. C) Soleus muscle force‐frequency relationship. *n* = 8 for all groups. D) Maximal specific isometric force in soleus developed during the test. *n* = 8 for all groups. E) Ca^2+^ amplitude (SR storage) upon 2.5 mm caffeine stimulation of FDB fibers, expressed as a percentage of the response of fibers isolated from BL10 mice. *n* = 10 for BL10, 11 for *mdx*, 12 for *mdx*+CHP. (F) SR Ca^2+^ uptake after 2.5 mm caffeine stimulation of FDB fibers, expressed as a percentage of the Ca^2+^ peak. *n* = 10 for BL10, 11 for *mdx* and *mdx*+CHP. A,C) Results represent the mean ± standard deviation. Two‐way ANOVA, followed by Dunnett's multiple comparison test versus *mdx* group, was used for statistical analysis. * BL10 compared to mdx; ^δ^ *mdx*+CHP compared to *mdx*. B,D,E,F) Whiskers in boxplots represent the min to max range. One‐way ANOVA, followed by Dunnett's multiple comparison test versus *mdx* group, was used for statistical analysis. *P* values are indicated as follows: ^*,δ^ *p *< 0.05; ^**,δδ^ *p *< 0.01.

Force generation in limb muscles was further tested ex vivo on the extensor digitorum longus (EDL) and soleus muscles, isolated from mice who underwent 17 weeks of treatment with CHP (study in Figure 1A). When tested for isometric contractions, muscles from *mdx* mice showed an overall lower force production and a downward shift of force‐frequency relationship compared to BL10 mice (**Figure** **2**A–D; Figure S1A,B, Supporting Information), as previously reported for this model.^[^ ^56^ ^]^ The contraction strength of EDL and soleus muscles was improved by CHP, with a partial recovery of the maximal force (Figure 2A–D; Figure S1A,B, Supporting Information), consistent with the preservation of muscle strength that was observed in vivo (Figure 1). EDL and soleus were further tested for their resistance against fatigue (Figure S1C,D, Supporting Information); however, fatigue resistance does not seem to be impaired in the *mdx* model, as also previously shown.^[^ ^56^ ^]^ When tested in an eccentric contraction setup, the impairment of *mdx* muscles was evident, but the treatment had no further compensating effect (Figure S1E, Supporting Information). Overall, ex vivo data confirmed the observed phenotype, supporting the fact that CHP preserves force production, but does not protect from eccentric injury.

Growing evidence suggests that the DAPC instability and the resulting damage in the sarcolemma, are also responsible for the disruption of Ca^2+^ homeostasis.^[^ ^57^, ^58^ ^]^ Ca^2+^ dysregulation is a common feature of fibers from DMD patients, and the *mdx* model mirrors this phenotype.^[^ ^59^ ^]^ Accordingly, *mdx* flexor digitorum brevis (FDB) muscle fibers showed sarcoplasmic reticulum (SR) Ca^2+^ depletion (Figure 2E; Figure S1F,G, Supporting Information). Additionally, Ca^2+^ uptake through store‐operated calcium channels after inducing SR Ca^2+^ depletion was generally upregulated in *mdx* (Figure 2F; Figure S1F,H, Supporting Information), likely as a compensatory mechanism for the constant SR Ca^2+^ depletion that occurs in this model.^[^ ^56^, ^57^, ^58^, ^59^, ^60^ ^]^ Fibers from mice treated with CHP not only had a higher SR Ca^2+^ content as compared to *mdx* muscle fibers but also store‐operated calcium entry (SOCE) was normalized (Figure 2E,F; Figure S1F–H, Supporting Information), indicating a better regulation of Ca^2+^ homeostasis.

### 2.3 CHP Restores DMD‐Induced Structural and Transcriptional Changes in Skeletal Muscle

**Figure 3:**
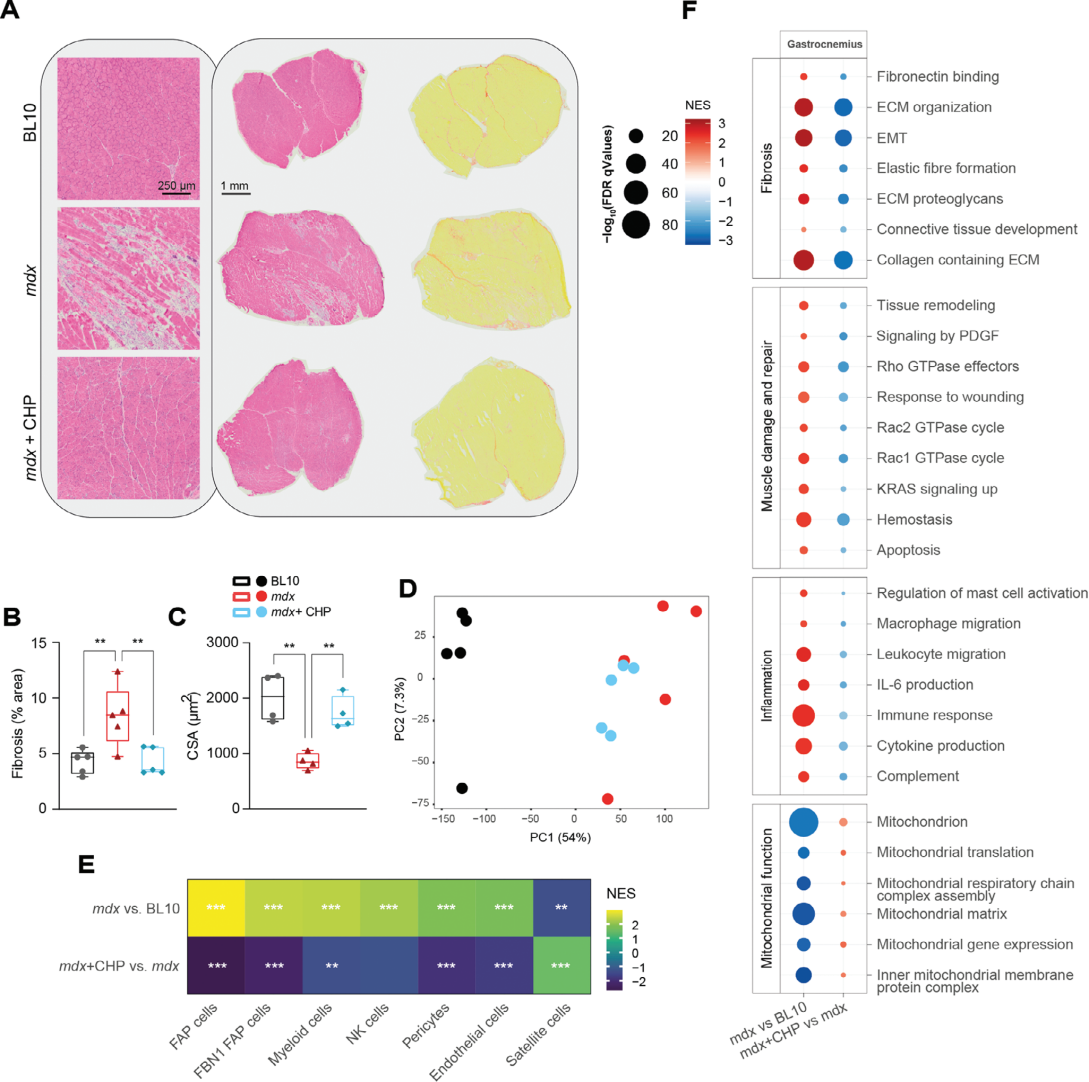
CHP decreases fibrosis and inflammation in the gastrocnemius muscle. A) Representative pictures of GM sections stained with hematoxylin and eosin (left) or Sirius red (right) to highlight fibrotic strands (in red). B) Quantification of Sirius red staining in histological images. *n* = 5. C) CSA of GM fibers, measured in histological sections stained with anti‐laminin antibody. *n* = 4 (>800 fibers analyzed per condition). B,C) Whiskers in boxplots represent the min to max range. One‐way ANOVA, followed by Dunnett's multiple comparison test versus *mdx* group, was used for statistical analysis. *p* values are indicated as follows: ^**^ *p *< 0.01; ^****^ *p *< 0.0001. D) PCA plot of the RNA‐seq profiles of the three groups. E) Cell type enrichment analysis of disease model (*mdx*) and treatment (CHP) effects on gene expression. *Q* values are indicated as follows: ^*^ *Q *< 0.05; ^**^ *Q *< 0.01; ^***^ *Q *< 0.001. F) Gene set enrichment analysis of disease model (*mdx*) and treatment (CHP) effects on gene expression. Gene sets are grouped into four categories: Fibrosis, Muscle damage and repair, Inflammation, and Mitochondrial function. CSA: cross‐sectional area. EMT: epithelial–mesenchymal transition. FAP: fibro‐adipogenic progenitor. FBN1: fibrillin‐1. IL‐6: interleukin 6. NK: natural killer. PDGF: platelet‐derived growth factor.

Gastrocnemius muscles (GMs) from *mdx* mice showed a marked deposition of connective tissue in the areas affected by muscle damage (**Figure** **3**A,B). Looking at muscle architecture, GM from *mdx* presented smaller fibers as a result of the cyclic degeneration‐regeneration process (Figure 3C). CHP reduced overall fibrosis and preserved muscle structure, as reflected in increased fiber size (Figure 3A–C). RNA sequencing was performed to further investigate the transcriptional changes in GM (Figure 3D). A cell‐type enrichment analysis suggested that treatment with CHP decreased fibro‐adipogenic progenitors (FAP), pro‐inflammatory cells, and cell populations involved in the response to tissue damage, while it preserved the population of satellite cells (Figure 3E). A gene set enrichment analysis (GSEA) was employed to evaluate the differential expression of pathways in the two comparisons *mdx* versus BL10 and *mdx*+CHP versus *mdx* (Figure 3F). GSEA revealed an increase in inflammation and fibrosis pathways in *mdx* muscles, which is typically associated with skeletal muscle degeneration in DMD (Figure 3F). Tissue damage and repair pathways such as apoptosis and tissue remodeling were also enhanced in DMD, coherent with the muscle fiber degeneration and regeneration that were already evident in the histological sections (Figure 3A,F). CHP down‐regulated the expression of pro‐inflammatory and pro‐fibrotic pathways, and reduced muscle damage‐associated pathways (Figure 3F). In a previous study, we described the positive effect of CHP on mitochondrial activity.^[^ ^45^ ^]^ In the GM, expression of mitochondrial pathways, including respiratory complexes and mitochondrial proteins, were enhanced by CHP (Figure 3F)—as opposed to a strong down‐regulation in *mdx* mice—suggesting a possible conserved mechanism of action of the compound in different tissues.^[^ ^45^ ^]^

### 2.4 CHP Attenuates the Development of Heart Fibrosis and Reduces Early Signs of a Modest Systolic Dysfunction

**Figure 4:**
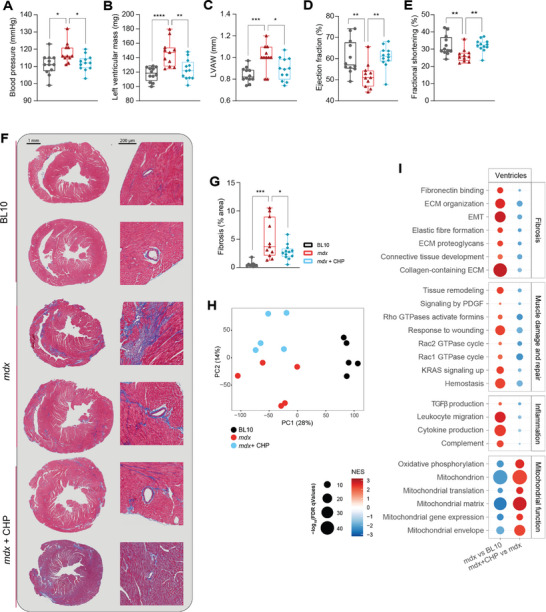
CHP prevented the development of the early signs of systolic dysfunction and heart fibrosis, decreased inflammation, and enhanced mitochondrial activity in *mdx* mice. A) Systolic blood pressure, measured at 18 weeks of age. *n* = 12 for BL10, 11 for *mdx*, 12 for *mdx*+CHP. B–G) Echocardiography was performed on BL10, *mdx* and *mdx* mice treated with CHP, at 20 weeks of age. *n* = 12 for BL10, 11 for *mdx*, 12 for *mdx*+CHP. B) Left ventricular mass. C) Left ventricular anterior wall (LVAW) thickness. D) Ejection fraction. E) Fractional shortening. F) Representative pictures of heart sections stained with Masson's trichrome to highlight fibrotic strands (in blue). On the left, full heart section; on the right, perivascular fibrosis. (G) Quantification of Masson's trichrome blue staining in histological images. *n* = 9 for BL10, 11 for *mdx*, 12 for *mdx*+CHP. A–E,G) Whiskers in boxplots represent the min to max range. One‐way ANOVA, followed by Dunnett's multiple comparison test versus *mdx* group, was used for statistical analysis. *p* values are indicated as follows: ^*^ *p *< 0.05; ^**^ *p *< 0.01; ^***^ *p *< 0.001; ^****^ *p *< 0.0001. H) PCA plot of the RNA‐seq profiles of the three groups. I) Gene set enrichment analysis of disease model (*mdx*) and treatment (CHP) effects on gene expression. Gene sets are grouped into four categories: Fibrosis, Muscle damage and repair, Inflammation, and Mitochondrial function. EMT: epithelial–mesenchymal transition. PDGF: platelet‐derived growth factor. TGFβ: transforming growth factor beta.

Cardiomyopathy represents a leading cause of death for DMD patients.^[^ ^14^, ^15^ ^]^ A first analysis of heart histology in the initial study (Figure 1A) highlighted the development of fibrotic patches in the cardiac muscle of *mdx* mice and suggested a potential prevention by CHP (Figure S2A, Supporting Information). Therefore, the development of cardiomyopathy and the protective effect of CHP on heart function were closely monitored in mice that followed the therapeutic protocol (Figure 1F). At 18 weeks of age, *mdx* mice displayed moderate hypertension, which was not observed in mice treated with CHP (**Figure** **4**A). Heart rate, as measured on conscious mice together with the blood pressure, was comparable between groups (Figure S2B, Supporting Information). An echocardiography was performed at 20 weeks of age to evaluate cardiac function and measure left ventricular myocardial thickness (Figure 4B–E). Compared to BL10 mice, *mdx* mice had evidence of ventricular hypertrophy (Figure 4B,C). These abnormal heart parameters in *mdx* mice were associated with a mild systolic dysfunction (decreased ejection fraction and fractional shortening), while diastolic function did not seem affected in this model (Figure 4D,E; Figure S2C,D, Supporting Information). Mice treated with CHP showed restored heart function, with normal systolic parameters and lower ventricular size, pointing to a therapeutic effect on DMD‐related early systolic dysfunction (Figure 4A–E; Figure S2B–D, Supporting Information).

The molecular and morphological alterations associated with DMD‐induced cardiovascular deterioration were further characterized by histology and RNA sequencing. Heart sections were stained with Masson's trichrome to visualize collagen deposition (in blue), highlighting the development of fibrosis in *mdx* mice, particularly evident around vessels (Figure 4F,G). The extension of fibrosis was reduced by CHP, confirming the anti‐fibrotic effect of the drug (Figure 4F,G). Ventricles from five mice for each group were randomly selected for transcriptomic analysis by RNA sequencing (Figure 4H). In the GSEA, ventricles from *mdx* mice showed a marked increase in pro‐fibrotic pathways, coherent with the histological results (Figure 4I). Pro‐inflammatory pathways were also positively enriched in *mdx* ventricles, as well as pathways related to apoptosis and tissue remodeling (Figure 4I). CHP was able to reduce the expression of pro‐fibrotic and pro‐inflammatory pathways, together with the response to tissue damage (Figure 4I). The antifibrotic and anti‐inflammatory effects of CHP could also be appreciated on the diaphragm (Figure S3A–C, Supporting Information), which is notably the tissue that is most affected by dystrophy in this model.^[^ ^53^, ^61^ ^]^ Proteins in the Ras homologous (Rho) GTPase subfamily—including Rho, Rac1, Rac2, Cdc42, ROCK—are known regulators of vascular smooth muscle cell contraction, and their activation plays a role in the development of cardiac hypertrophy, systemic hypertension, and other cardiovascular diseases.^[^ ^62^, ^63^, ^64^, ^65^ ^]^ Pathways associated with Rho factor signaling were also up‐regulated in the ventricles of the *mdx* model (Figure 4I). Supporting the therapeutic role of CHP in DMD‐associated cardiovascular dysfunction, tissue remodeling, and Rho‐related pathways were down‐regulated by the treatment (Figure 4I). In the cardiac muscle, CHP also enhanced the expression of genes linked to mitochondrial activity coherent with the transcriptomic changes observed in GM (Figure 4I), suggesting that the therapeutic properties of CHP might rely on the same mechanisms of action in both muscle types.

### 2.5 Prolonged Treatment with CHP Effectively Delayed Age‐Related Sarcopenia

**Figure 5:**
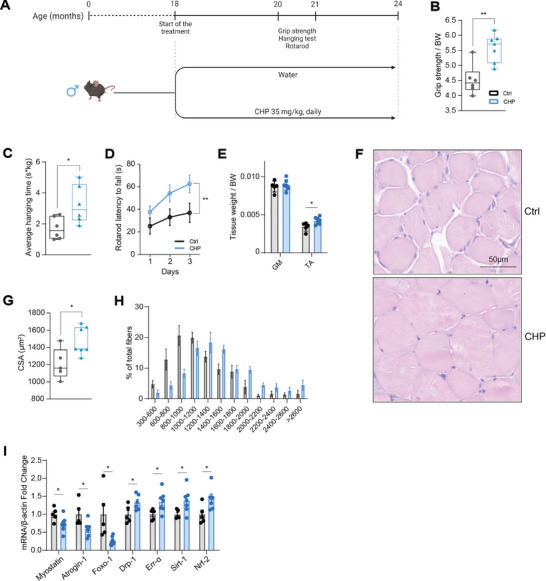
Prolonged treatment with CHP prevented muscle deterioration in aging. A) Schematic representation of the animal experiment. Aged C57BL6/J mice were treated with CHP (35 mg kg^−1^) from months 18 to 24. Grip test, hanging test, and rota‐rod were performed as indicated. B) Grip strength normalized on BW. Whiskers in boxplots represent the min to max range. *n* = 6 for Ctrl, 7 for CHP. C) Average hanging time on a hanging exercise normalized on BW. *n* = 6. Whiskers in boxplots represent the min to max range. *n* = 6. D) Rotarod test performance over 3 consecutive days. Data are shown as mean ± SEM. *n* = 6 for Ctrl, 7 for CHP. E) Tissue weight of GM and TA normalized on the BW. Data are shown as mean ± SD. *n* = 5 for Ctrl, 7 for CHP. F) Representative pictures of GM stained with hematoxylin and eosin. G) CSA of GM fibers, measured in histological sections stained with hematoxylin and eosin. Whiskers in boxplots represent the min to max range. *n* = 5 for Ctrl, 7 for CHP (>500 fibers analyzed per condition). H) Histogram of CSA distribution of GM muscle fibers. Data are shown as mean ± SEM. I) Gene expression in GM. Data are shown as mean ± SEM. *n* = 5 for Ctrl, 7 for CHP. Unpaired t‐test (B,C,E,G,I) or two‐way ANOVA (D) were used for statistical analysis. *P* values are indicated as follows: ^*^ *p *< 0.05; ^**^ *p *< 0.01. A) Created with BioRender.com.

We then tested the therapeutic effects of CHP on age‐related muscle decline. Aged C57BL6/J mice were started on CHP at 18 months of age, given daily doses of 35 mg kg^−1^ for a total of 6 months (**Figure** **5**A). Notably, a few mice died in the control group, while none of the aged mice in the treated group were lost (Figure S4A, Supporting Information), possibly indicating a positive effect of CHP on lifespan extension. In agreement, CHP treatment extended lifespan in wild‐type *C. elegans* (Figure S4B, Supporting Information); further evaluation is warranted to understand the effect of CHP on lifespan. In the aging mouse model, CHP improved muscle function, as evidenced by increased strength in grip and hanging tests (Figure 5B,C). CHP‐treated aged mice also exhibited better performance in the rotarod test (Figure 5D), suggesting enhanced motor coordination, perhaps mediated through an effect on the central nervous system. Among the muscles analyzed, tibialis anterior (TA) increased in size upon CHP, while the weight of GM was unchanged (Figure 5E). Nonetheless, histological analysis of the GM revealed a significant increase in the cross‐sectional area (CSA) of muscle fibers in the treated cohort (Figure 5F–H). These structural changes were accompanied by the down‐regulation of genes associated with the development of muscular atrophy during aging (*myostatin, atrogin‐1, foxo‐1, dystrophin*) (Figure 5I).^[^ ^66^, ^67^, ^68^, ^69^ ^]^ Moreover, CHP substantially increased the expression of *nrf‐2*, a well‐known master regulator of redox homeostasis and antioxidant capacity^[^ ^70^ ^]^ (Figure 5I). These findings demonstrate that CHP effectively delayed muscle aging, mitigated functional decline, and improved overall muscle function potentially through transcriptional remodeling.

**Figure 6:**
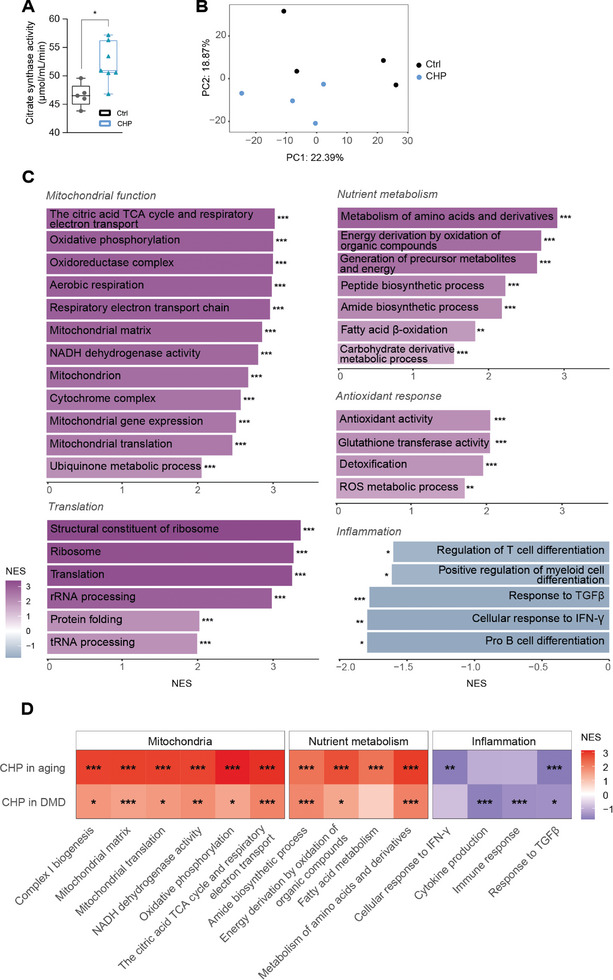
CHP preserved muscle mitochondrial function and cellular metabolism in aged mice. A) Citrate synthase activity in TA muscle. Whiskers in boxplots represent the min to max range. *n* = 5 for Ctrl, 7 for CHP. An unpaired t test was used for statistical analysis. ^*^ *p *< 0.05. B) PCA plot of the RNA‐seq profiles of the two groups. C) Gene set enrichment analysis of CHP effects on the gene expression of TA. Gene sets are grouped into five categories: Mitochondrial function, Translation, Nutrient metabolism, Antioxidant response, and Inflammation. D) Heatmap comparing the GSEA results between aging and DMD models for select pathways. Results are presented as a comparison between the treated (with CHP) and untreated disease models. *Q* values are indicated as follows: ^*^ *Q *< 0.05; ^**^ *Q *< 0.01; ^***^ *Q *< 0.001. IFN‐γ: interferon gamma. ROS: reactive oxygen species. TCA: tricarboxylic acid. TGFβ: transforming growth factor beta.

To further elucidate the underlying mechanisms responsible for these positive effects, we evaluated mitochondrial function. Recent reports have emphasized the potential of CHP to enhance mitochondria biogenesis and function.^[^ ^45^, ^71^ ^]^ Citrate synthase activity, which serves as a reliable marker for mitochondrial density and function in skeletal muscle, was increased in the TA of these aged CHP‐treated animals (**Figure** **6**A). RNA sequencing was performed on TA samples to highlight the pathways differentially regulated by the treatment (Figure 6B). The activation of pathways related to mitochondrial homeostasis was evident, marked by increased expression of complexes within the respiratory chain and its structural components (Figure 6C). Pathways involved in nutrient metabolism and energy regulation were also upregulated (Figure 6C), suggesting that CHP counteracts the metabolic changes and the decrease in metabolic rate that naturally occurs with aging.^[^ ^72^, ^73^, ^74^, ^75^ ^]^ Antioxidant response and detoxification processes were also enhanced consistent with the increased *Nrf2* transcript levels (Figure 6C). Interestingly, among the top hits of the unbiased GSEA, CHP upregulated several pathways related to translation and ribosome structure (Figure 6C), which are notably impaired in aging.^[^ ^76^, ^77^, ^78^, ^79^ ^]^ CHP also confirmed its known anti‐inflammatory effect (Figure 6C). In an effort to delineate the molecular effects of CHP on skeletal muscle, we compared the GSEA from the DMD and sarcopenia study (Figure 6D). This comparison indicated that CHP improved muscle function in both settings by enhancing the expression of genes involved in mitochondria biogenesis and metabolism, while inhibiting pro‐inflammatory pathways (Figure 6D). This suggests the pivotal role of these processes for healthy aging, underlying the therapeutic effects of CHP in promoting muscle health and counteracting age‐related functional decline.

## 3 Discussion

Currently, very few compounds are reported to be capable of improving muscle function in humans in monogenic as well as age‐related muscle disorders. Identifying new treatment strategies that preserve and/or improve muscle function is hence an absolute priority. Here we report that CHP can improve muscle function in both mouse models of DMD, an inherited muscle disease, and age‐related sarcopenia. The field of DMD research is rapidly advancing toward understanding DMD pathogenesis, resulting in new FDA‐approved therapies to restore the functionality of dystrophin.^[^ ^18^, ^19^ ^]^ Nevertheless, current protocols for managing DMD symptoms and delaying disease progression, which will likely be required in combination with gene therapy, come with many side effects.^[^ ^21^, ^22^ ^]^ On the other hand, sarcopenia was only recognized as a disease in 2016, and drug development for this condition is still at the early stages.^[^ ^28^ ^]^ Mitochondrial modulators such as mitophagy enhancers (Urolithin A^[^ ^38^, ^39^, ^40^, ^41^ ^]^) and nicotinamide adenine dinucleotide boosters (such as nicotinamide mononucleotide^[^ ^80^ ^]^ or nicotinamide riboside^[^ ^81^ ^]^) have emerged as strategies to manage sarcopenia, but there is still a substantial unmet medical need, as the pharmacological profile of these compounds is still being improved.

Here, we provide evidence that CHP has a promising pharmacological profile to manage specific aspects of both types of muscle disorders. CHP is safe for human administration, and initial clinical studies have not reported any significant side effects (NCT02784275; NCT03560271). In a recent publication, we identified CHP as an anti‐inflammatory and antifibrotic agent in models of liver disease. Additionally, we demonstrated that the transcriptomic signature of CHP extends beyond the liver, influencing similar gene regulatory networks in a systemic fashion.^[^ ^45^ ^]^

Among the first symptoms of DMD, children show reduced mobility and muscle weakness, as the disease progressively affects limb muscles.^[^ ^2^ ^]^ In the *mdx* mouse model of DMD, this was reflected in the loss of strength and exercise endurance, visible also as decreased contractility and resistance to eccentric damage when muscle fibers were tested ex vivo. CHP treatment restored force generation and maximal strength of the fibers, both in vivo and ex vivo. While a better Ca^2+^ handling, which is notably impaired in DMD,^[^ ^57^, ^58^, ^59^ ^]^ can play a role in the process, this can also be explained by other mechanisms such as alterations in myofibrillar Ca^2+^ sensitivity and/or in the force‐generating capacity at the cross‐bridge level. However, the treatment did not improve endurance in downhill running exercise protocols, nor protected the fibers against eccentric injury, suggesting that CHP was not effectively reducing the frailty of the sarcolemma. This indicates that these two aspects of DMD pathogenesis, decreased fiber strength and sarcolemma weakening, are uncoupled and not necessarily co‐dependent, suggesting that different therapeutic approaches might be needed to recover both.

The antifibrotic action of CHP, first observed in the liver,^[^ ^45^ ^]^ was also confirmed in this study, showing attenuation of the development of fibrosis in skeletal and cardiac muscle. Heart failure represents a leading cause of death for DMD patients, who ultimately develop cardiomyopathy after the accumulation of damage and fibrosis in the cardiac muscle.^[^ ^14^, ^15^ ^]^ The *mdx* mouse is known to develop cardiomyopathy as it ages, although the age at which this model develops the first signs of disease is not coherent across studies.^[^ ^52^, ^82^, ^83^, ^84^, ^85^, ^86^, ^87^ ^]^ In our experiment, we were able to highlight early signs of an abnormal cardiac phenotype. In the *mdx* model, the heart showed marked fibrosis, which was associated with ventricular hypertrophy and mild systolic dysfunction. Tissue damage was highlighted in the ventricular transcriptome, together with the rise in pro‐inflammatory pathways. CHP was able to counteract the development of fibrosis, preventing heart hypertrophy and decreasing inflammation, hence improving overall cardiac function as evaluated by echocardiography. Interestingly, the transcriptomic signature of CHP in ventricles was consistent with the one observed in the GM, suggesting once again a systemic action of the compound. The beneficial effects of CHP on the heart and diaphragm strengthen the importance of future clinical investigations, as delaying the onset of cardiomyopathy and the spread of dystrophy to respiratory muscles might be critical to extending the life expectancy of DMD patients. Furthermore, future studies aiming at evaluating the effects of CHP in other types of cardiomyopathies that are typified by fibrosis, for example seen after myocardial infarction, are needed to unveil other therapeutic applications.

CHP has also proved to be well‐tolerated and effective as a long‐term treatment in a mouse model of age‐related sarcopenia. This treatment prevented the progressive muscle atrophy that characterizes the aging process, thereby preserving muscle structure and functionality. Moreover, CHP reversed the transcriptional changes commonly associated with aging, preserving muscle metabolic function and antioxidant response. Importantly, CHP enhances mitochondrial function, which is profoundly impaired with aging.^[^ ^88^, ^89^ ^]^ Maintaining muscle mitochondrial function has been proven to be beneficial in the context of several diseases, including DMD^[^ ^38^, ^39^, ^90^ ^]^ and aging.^[^ ^88^, ^91^, ^92^ ^]^

We acknowledge the lack of a clear mechanism of action for CHP as the main limitation of this study. Although further work is required to fully characterize the molecular targets of CHP, we believe that the positive impact of CHP on muscle function coupled with its large therapeutic index in humans provides a solid basis for testing CHP as a potential new treatment to manage the symptoms of DMD and delay the onset of sarcopenia and its associated comorbidities.

## 4 Experimental Section

### Animal Studies

C57BL/10ScSn (BL10) and C57BL/10ScSn‐*Dmd^mdx^* (*mdx*) male mice (The Jackson Laboratory) were housed at 22 °C with free access to water and chow diet ad libitum. After a week of adaptation, mice were divided into three groups: *mdx* received either CHP treatment or water (control), and BL10 mice received water (control), three times per week by oral gavage. For the preventive treatment protocol, mice were treated from 3 to week 20 of age with CHP at a dose of 20 mg kg^−1^ or water as control. The following tests were performed: grip test at week 10, uphill running at week 12 and downhill running at week 18. For the therapeutic treatment protocol, mice were treated from 7 to week 22 of age with CHP at a dose of 35 mg kg^−1^ or water as control. The following tests were performed: hanging test at week 15, grip test at week 16, noninvasive blood pressure (NIBP) at week 18, and echocardiography at week 20. At the end of the studies, mice were euthanized to collect tissues. For biochemical analysis, tissues were collected and flash‐frozen, and stored at −80 °C.

18‐month‐old male C57BL6/J mice (Korea basic science institute [KBSI]) were housed in individual cages in constant temperature and humidity air‐conditioning cabinet (JD‐SY‐02, Jungdo) at a temperature of 23 ± 3°C and a humidity of 50% with a 12 hours light/dark cycle. Mice were free to access distilled water (DW) and laboratory chow diet ad libitum. Mice were divided into two groups based on equal average body weight, and DW or CHP (35 mg kg^−1^/day) was orally administered for 6 months. Further detailed information on the phenotyping tests can be found in supplementary methods.

### Ex Vivo Muscle Force Assessment

Muscle mechanical measurements were assessed as previously described^[^ ^93^ ^]^ with slight modifications. All assays were measured in a blinded fashion. BL10, *mdx*, and CHP‐treated *mdx* mice were euthanized by cervical dislocation. EDL and soleus muscles were quickly dissected, then bathed in a 10 mL horizontal chamber containing a continuously oxygenated Krebs solution composed of 135.5 mm NaCl, 5.9 mm KCl, 1 mm MgCl_2_, 2 mm CaCl_2_, 11.6 mm HEPES sodium, and 11.5 mm glucose, pH 7.4 at 25 °C. The muscle was tied between a dual‐mode lever arm and a fixed hook, and stimulation was delivered through platinum electrodes running parallel to the muscle (1500A Intact Muscle Test System, Aurora Scientific Inc., Canada). Resting muscle length (L_0_) was carefully adjusted for the maximal isometric force with 125‐Hz maximally fused tetani. The force‐frequency relationship was determined by sequentially stimulating the muscles with 25, 50, 75, 100, 125, and 150 Hz stimulation trains of 300 ms duration with 1 min rest between each contraction. Muscle‐specific force was obtained by normalizing force data by the CSA (mN mm^−2^), calculated by dividing muscle blotted weight (mg) by the product of fiber length and 1.06 g cm^−3^, the density of mammalian skeletal muscle, the fiber length being equal to 0.45 x L_0_ (determined muscle resting length) for EDL and 0.7 x L_0_ for soleus.^[^ ^94^ ^]^ To investigate muscle fatigue resistance, muscles were subjected to 125 Hz stimulation trains of 300 ms duration at 10 s intervals over 50 s for EDL muscles and at 1 s intervals over 120 s for soleus muscles.^[^ ^56^ ^]^ Data from each experiment were analyzed with Aurora's DMA software (Aurora Scientific Inc., 2002, Solwood Enterprises, Inc., 2002) and Microsoft Excel.

### Ex vivo EDL Muscle Eccentric Contractions

The eccentric contractions were performed as previously described.^[^ ^95^ ^]^ Briefly, EDL muscles were subjected to a series of seven eccentric contractions consisting in 500 ms tetani during which a stretch of 1 mm was applied 160 ms after the start of stimulation and maintained up to 250 ms after the start of stimulation (10 s interval between two consecutive tetani). Isometric force was measured for each tetanus just before the onset of the stretch and the percentage of force drop was calculated.

### Ca^2+^ Imaging Using Fluo‐4 AM in FDB Muscle Fibers

FDB muscle fibers were loaded with the cytosolic Ca^2+^ indicator Fluo‐4/AM (5 µm, Invitrogen, Basel, Switzerland) solubilized in a Krebs Ca^2+^solution (in mM: NaCl 135.5, MgCl_2_ 1.2, KCl 5.9, glucose 11.5, HEPES 11.5, CaCl_2_ 1.8 (pH 7.3)) for 20 min in the incubator, then rinsed twice with Krebs solution. For caffeine stimulation, fibers were washed twice and kept in the Krebs Ca^2+^ solution. Fluo‐4 fluorescence was monitored using a confocal microscope system (Zeiss LSM 5 Live, 40x oil immersion lens, excitation wavelength was 488 nm and the emitted fluorescence was recorded between 495 and 525 nm) in a time‐lapse acquisition framework. After recording the basal fluorescence, fibers were stimulated with 2.5 mm caffeine (final concentration) (O1728‐500, Thermofisher Scientific) to deplete SR Ca^2+^ stores. For the SOCE measurements, immediately prior to image acquisition, fibers were washed twice with Ca^2+^‐free Krebs solution and then kept in Ca^2+^‐free Krebs solution. After recording the basal fluorescence, fibers were stimulated with 1 µm thapsigargin (final concentration) (Tg, T9033, Thermofisher Scientific) to trigger Ca^2+^ release from the SR. 2 mm CaCl_2_ was finally applied to assess the SOCE. Zen software (products/microscopy‐software/zenlite/zen‐2‐lite) was used for the acquisition and the data were extracted into Excel files for analysis. The use of the single excitation/emission dye fluo‐4 necessitates normalizing to prestimulation values to account for possible differences in dye loading.^[^ ^96^ ^]^ The amplitude of SR Ca^2+^ stores was calculated by subtracting the peak fluorescence from the baseline. The amplitude of the Ca^2+^ transient plateau was calculated and expressed as a percentage of the SR Ca^2+^ peak amplitude and indirectly reflects the contribution of the SOCE. The actual SOCE evaluated with Tg stimulation was calculated as the difference between the Ca^2+^ amplitude after adding 2 mm CaCl_2_ and the smallest Ca^2+^ levels before adding CaCl_2_. These smallest Ca^2+^ levels before CaCl_2_ addition were used to estimate the Ca^2+^ reuptake levels (calculated as a percentage of the SR Ca^2+^ peak).

### Histology

For histological analysis of tissues from the DMD study, heart samples were washed with PBS (Gibco, 10010023) and fixed in 10% formalin (ThermoFisher, 9990244). After 24 h, samples were embedded in paraffin blocks. 4 µm paraffin sections were processed with standard Masson's trichrome staining to highlight collagen fibers. GM and diaphragms were harvested and enclosed in Tragacanth gum (G1128, Sigma), then froze for 1 min in isopentane that was precooled with liquid nitrogen. For GM, 8 µm cryosection sections were processed with standard hematoxylin and eosin, Sirius red protocols or immunostained for laminin (L9393, Sigma‐Aldrich). Diaphragms were stained with standard Masson's trichrome or immunostained for CD45 (rat α‐CD45, Thermo Fisher). Images were taken with an Olympus Slide Scanner VS120 L100 at 40x magnification. Digital slides were analyzed using QuPath software.^[^ ^97^ ^]^ CSA and areas affected by fibrosis and inflammation were quantified using ImageJ‐Fiji software.^[^ ^98^ ^]^ For aged mice, GM was fixed in a neutral buffered 10% formalin solution (HT‐501128, Sigma) and embedded in paraffin. Thin sections were processed to be hydrated and then stained with hematoxylin and eosin methods. The stained sections were imaged using a microscopy (Olympus BX53 upright microscope, Olympus, Tokyo, Japan). 4–5 images were taken per mouse, and the CSA of each muscle fiber was measured using ImageJ software.

### RNA‐Seq

For the DMD study, RNA was extracted in Trizol (TriPure, Roche) from frozen ventricles or GM tissue, using the NucleoSpin RNA extraction kit (Macherey‐Nagel), and bulk RNA‐seq was performed by the Beijing Genomics Institute with the BGISEQ‐500 platform. From age mice, total RNA was extracted from GM using NucleoZOL reagent (740404.200, Macherey‐Nagel), and paired‐end sequencing was performed by Macrogen Incorporated with Illumina NovaSeq. Clean reads were obtained by removing adapter sequences or low‐quality sequences (RIN<8) based on SOAPnuke software.^[^ ^99^ ^]^ RNA‐seq data and related analyses were performed using the R version 4.1.0.^[^ ^100^ ^]^ All samples passed the quality check by FastQC.^[^ ^101^ ^]^ Sequences were aligned against the mouse genome, using STAR (version 2.6.0a).^[^ ^102^ ^]^ Three comparisons (*mdx* versus BL10, *mdx*+CHP versus *mdx*, aged+CHP versus aged) were analyzed by differential expression analysis using the limma R package (version 3.50.3).^[^ ^103^, ^104^ ^]^ GSEA was performed from genes sorted by ‐log_10_(p‐value) *sign(logFC), using clusterProfiler R package (version 4.2.2),^[^ ^105^ ^]^ using gene sets (Reactome, GO, Hallmarks, and KEGG) retrieved with the msigdbr R package (version 7.5.1).^[^ ^106^, ^107^, ^108^ ^]^ The gene sets with absolute normalized enrichment score (NES) higher than one and false discovery rate (qValue) lower than 0.05 are identified as significantly enriched gene sets. Cell‐type enrichment analysis was performed analogously using the MSigDB cell type signature gene sets for muscle cells.

### Data Analysis and Statistics

GraphPad Prism 9.3.1 was used for all statistical analyses. Differences between the groups were assessed using either one‐way/two‐way analysis of variance (ANOVA)—followed by Dunnett's multiple comparison test—either unpaired *t*‐test, as specified in figure legends. All *p* values <0.05 were considered significant. ^*^ *p *< 0.05; ^**^ *p *< 0.01; ^***^ *p *< 0.001; ^****^ *p *< 0.0001. Sample size (n) for each statistical analysis is indicated in each figure legend. Differences in n are due to mice loss during the experiment, or to the exclusion of mice with behavioral despair/anxiety from tests in which behavior negatively affected performance (e.g., running performance, hanging test).

### Ethics Approval

All animal experiments were carried out following Swiss ethic guidelines and were authorized by the animal experimentation committee of the Canton de Vaud (DMD study, VD2665) or approved in accordance with the Ethics Review Committee of the Pohang Advanced Bio Convergence Center, Republic of Korea (sarcopenia study, ABCC2022016).

## Conflict of Interest

This work was in part funded by a grant from NovMetaPharma. D.L. and J.J. are employees of NovMetaPharma. H.Y.J. is a board member of NovMetaPharma. J.A. is a founder and/or consultant to MitoBridge/Astellas, Metro Biotech, Amazentis, Vandria, Orso Bio, and NovMetaPharma. All other authors declare that they have no competing interests.

## Author Contributions

N.Z., K.S., and D.L. contributed equally to this work. A.D.M., H.Y.J., and J.A. conceived the study. A.D.M., D.L., and H.Y.J. performed the in vivo experiments and the analysis of the experimental results. N.Z. and A.D.M. performed ex vivo measurements. A.D.M., D.L., and J.J. performed histological and molecular analyses. K.S. and X.L. carried out RNA sequencing analysis. A.D.M., H.Y.J., and J.A. wrote the manuscript with contributions from all co‐authors. J.A. and H.Y.J. supervised the study and secured funding.

## Supporting information

Supporting Information

## Data Availability

The data that support the findings are available upon request to the corresponding author (J.A.). RNA‐seq data are available on Gene Expression Omnibus (GEO accession number GSE241771). Methods, materials, and resources are included in the Materials and Methods or Supplementary Methods.
