# Supplementary material for: Cyclo His‐Pro Attenuates Muscle Degeneration in Murine Myopathy Models
Source: Adv Sci (Weinh). 2024 May 10;11(28):2305927. doi: 10.1002/advs.202305927 (PMC11267275; doi:10.1002/advs.202305927)
Supplement: Supplementary file 1 — Supporting Information [file ADVS-11-2305927-s001.docx]

**Supplementary material**

**Cyclo His-Pro attenuates muscle degeneration in murine myopathy models**

Alessia De Masi^1^, Nadège Zanou^2, #^, Keno Strotjohann^1, #^, Dohyun Lee^3, #^, Tanes I. Lima^1^, Xiaoxu Li^1^, Jongsu Jeon^3^, Nicolas Place^2^, Hoe-Yune Jung^3, 4,^ *, Johan Auwerx^1,^ *.

^1^ Laboratory of Integrative Systems Physiology, Institute of Bioengineering, École Polytechnique Fédérale de Lausanne, Lausanne 1015, Switzerland

^2^ Institute of Sport Sciences and Department of Biomedical Sciences, Faculty of Biology-Medicine, University of Lausanne, Lausanne, Switzerland.

^3^ R&D Center, NovMetaPharma Co., Ltd., Pohang, 37668, South Korea

^4^ School of Interdisciplinary Bioscience and Bioengineering, Pohang University of Science and Technology (POSTECH), Pohang, 37673, South Korea

^#^ These authors contributed equally.

* Corresponding authors:

Johan Auwerx – Address: Laboratory of Integrative and Systems Physiology, Ecole Polytechnique Fédérale de Lausanne, CH-1015 Lausanne, Switzerland. Tel.: +41 216939522. E-mail address: admin.auwerx@epfl.ch

Hoe-Yune Jung – Address: R&D Center, NovMetaPharma Co., Ltd., Pohang, 37668, South Korea. Tel.: +82 (0)54 223 2893. E-mail address: elijah98@novmeta.com

Table of contents

[**Supplementary methods** 2](#_Toc152339746)

[**Supplementary figures** 4](#_Toc152339747)

[**Supplementary tables** 6](#_Toc152339748)

# **Supplementary methods**

*Grip test*. Muscle strength was evaluated by grip test as previously described [1].

*Uphill running*. Mice were familiarized with the treadmill setting the day prior the experiment. Uphill running was performed on a treadmill with an inclination of +5°. The exercise started at a speed of 9 cm/s and was gradually increased of 3 cm/s every 20 minutes. The exhaustion endpoint was considered reached when mice accumulated at least 5 shocks (0.1 mA) per minute for two consecutive minutes. The maximal duration of the experiment was 3 hours. The distance traveled and time before exhaustion were measured.

*Downhill running*. Mice were familiarized with the treadmill setting the day prior the experiment. Downhill running was performed on a treadmill with an inclination of -5°. The exercise started at a speed of 9 cm/s and was gradually increased of 3 cm/s every 12 minutes. The exhaustion endpoint was considered reached when mice accumulated at least 5 shocks (0.1 mA) per minute for two consecutive minutes. The maximal duration of the experiment was 90 minutes. The distance traveled and time before exhaustion were measured.

*Hanging test*. Mice were acclimated to the testing room 30 minutes prior to testing. The animal was placed on a wire grid, grasping the grid with all four limbs. The grid was then flipped over so that the animal was hanging, and the time that the mouse was able to hold onto the grid was measured. The maximum trial length was 2 minutes and 30 seconds (150s). The latency to fall was measured five times for each mouse, with 10 minutes intervals in between trials.

*Rotarod test.* Motor coordination and balance were measured using an accelerating rota-rod (Panlab, Harvard Apparatus, Spain). Mice were placed on a rotating rod and recorded the time until they fall from the rod. Each mouse was given 3 trials per day with accelerating rotation from 4 to 40 rpm during 300 sec. The three recorded latencies to fall each day were averaged.

*Non-invasive blood pressure*. Systolic blood pressure and heart rate were measured by a computerized tail-cuff system (BP-2000, Visitech Systems, Apex, NC) in conscious animals. Measurements were made at fixed diurnal interval. During the measurement, the device was maintained at 36°C to ensure a good blood flow in the tail artery. Each measurement unit comprised of one inflatable cuff, one photo-emitter LED and one photo-electric sensor. Each mouse was placed in a dark individual restrainer, with the tail placed through the inflatable cuff and attached onto the sensor. Following 10 preliminary measurements to accustom mice to the procedure, 10 actual measurement cycles were collected on 5 consecutive days and averaged for each individual animal.

*Echocardiography*. Mice were anesthetized by inhalation of isoflurane for 30-45 minutes and shaved for the procedure. Mice were placed in a ventrodorsal position on the heated pad set to 37°C in order to maintain the body temperature. Echocardiography was performed to assess cardiac function using a 10 MHz linear transducer and a cardiovascular ultrasound system (Vevo 2100, VisualSonics). The left ventricular (LV) end-systolic dimension (LVDs) and end-diastolic dimension (LVDd) were measured in parasternal short axis (PSAX) view in M-mode tracings at the midpapillary level. The LV end-diastolic volume (LVEDV), the LV end-systolic volume (LVESV), the ejection fraction (EF) and fractional shortening (FS) were measured to examine the systolic function. Isovolumic relaxation time (IVRT), normalized on heart rate, and the ratio of the early [E] to late [A] ventricular filling velocities were assessed in pulsed-wave doppler mode.

*EchoMRI.* Non-invasive monitoring of fat and lean mass using Echo Medical Systems as previously described [2]. EchoMRI was performed at week 3 and 14 for the preventive study, at week 7, 14, 21 for the therapeutic study. Results are reported in Tables S1 and S2.

*qPCR.* Total RNA was extracted from TA using NucleoZOL reagent (740404.200, Macherey-Nagel). 1μg of total RNA was used for cDNA synthesis using ReverTra Ace qPCR RT Master Mix (Toyobo, Japan). Real-time qPCR (RT-qPCR) was performed using SYBR Green Realtime PCR Master mix (Toyobo, Japan) and primer sets (Table S3). Gene expression levels were normalized to those of β-actin.

*Citrate Synthase Activity.* Citrate synthase activity was measured using a commercial assay kit (Sigma, CS0720) as the manufacturer’s protocol. Briefly, TA muscle tissue extracts were incubated with oxaloacetate and acetyl-CoA in a suitable buffer at 37 °C to generate CoA-SH, a thiol subsequently detected by a reaction with 5,5′-dithiobis (2-nitrobenzoic acid) to produce absorbance at 412 nm. Enzymatic activity was normalized on protein content.

*C. elegans* lifespan experiment. The Bristol strain (N2, Caenorhabditis Genetics Center [CGC]) was used as the wild-type strain. The experiment was performed at 20°C as previously described [3]. Briefly, 80–100 worms per condition were plated on Nematode Growth Medium plates containing vehicle (water) or CHP 2.5 mM, and seeded with E. coli strain HT115 (CGC). Treatment with CHP started at maternal L4 stage. Worms were scored three times a week, and transferred to fresh plates every week.

*Figures.* BioRender was used to draw the animal studies outlines. Graphs were created with GraphPad Prism 9.5.1. Adobe Illustrator 26.0.1 was used to assemble figure panels.

[1] P. P. Laurila, P. Luan, M. Wohlwend, N. Zanou, B. Crisol, T. I. de Lima, L. J. E. Goeminne, H. Gallart-Ayala, M. Shong, J. Ivanisevic, N. Place, J. Auwerx, *Sci. Adv.*, **2022**, vol. 8, no. 4, p. 4423, doi: 10.1126/SCIADV.ABH4423.

[2] C. A. Argmann, M. Champy, J. Auwerx, *Curr. Protoc. Mol. Biol.*, **2006**, vol. Chapter 29, no. 1, doi: 10.1002/0471142727.MB29B01S73.

[3] R. H. Houtkooper, L. Mouchiroud, D. Ryu, N. Moullan, E. Katsyuba, G. Knott, R. W. Williams, J. Auwerx, *Nature*, **2013**, doi: 10.1038/nature12188.

# **Supplementary figures**


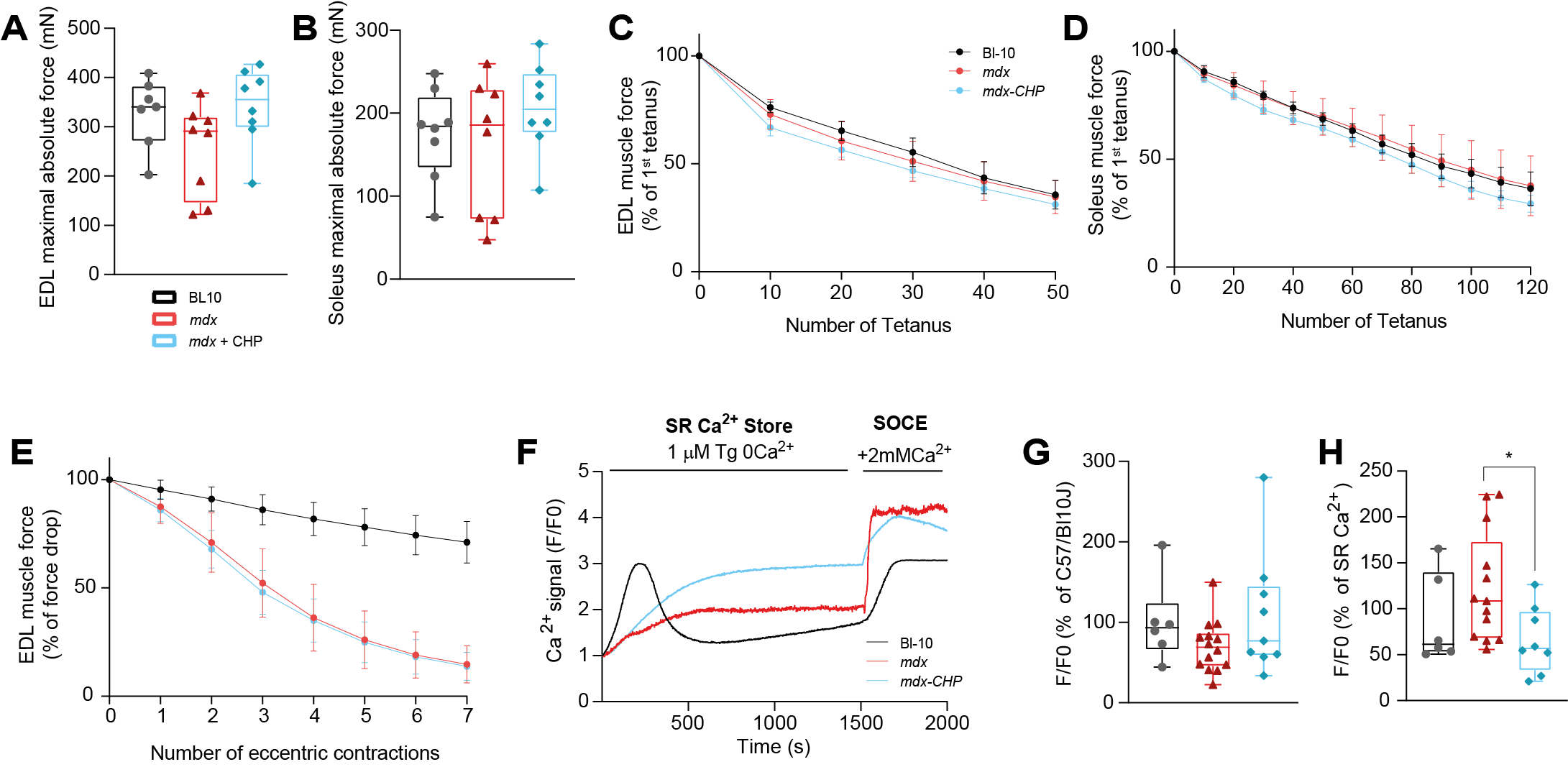


**Figure S1. Fatigue resistance is not affected in mdx mice, but muscle fibers are prone to eccentric injury.** (A) Maximal absolute isometric force in EDL developed during the test. n=7 for BL10, 8 for mdx and mdx+CHP. (B) Maximal absolute isometric force in soleus developed during the test. n=8 for all groups. (C) Quantification of the EDL muscle force produced during the fatigue protocol (force quantified every 10th tetanus). (D) Quantification of the soleus muscle force produced during the fatigue protocol (force quantified every 10th tetanus). (E) Quantification of the EDL muscle force produced during the eccentric contraction protocol. (F) Ca^2+^ traces in FDB fibers after stimulation with 1 μM thapsigargin and following 2mM Ca^2+^ administration. (G) Ca^2+^ amplitude (SR storage) after 1 μM thapsigargin stimulation of FDB fibers, expressed as percentage of the response of fibers isolated from BL10 mice. n=6 for BL10, 14 for mdx, 9 for mdx+CHP. (H) SOCE quantification: SR Ca^2+^ uptake after 1 μM thapsigargin stimulation of FDB fibers, expressed as percentage of the peak of Ca^2+^ release. n=6 for BL10, 13 for mdx, 8 for mdx+CHP. Whiskers in boxplots represent the min to max range (A, B, G, H); results represent the mean ± standard deviation (C-E). One-way ANOVA, followed by Dunnett’s multiple comparison test versus mdx group, was used for statistical analysis (A, B, G, H). * *P*<0.05.


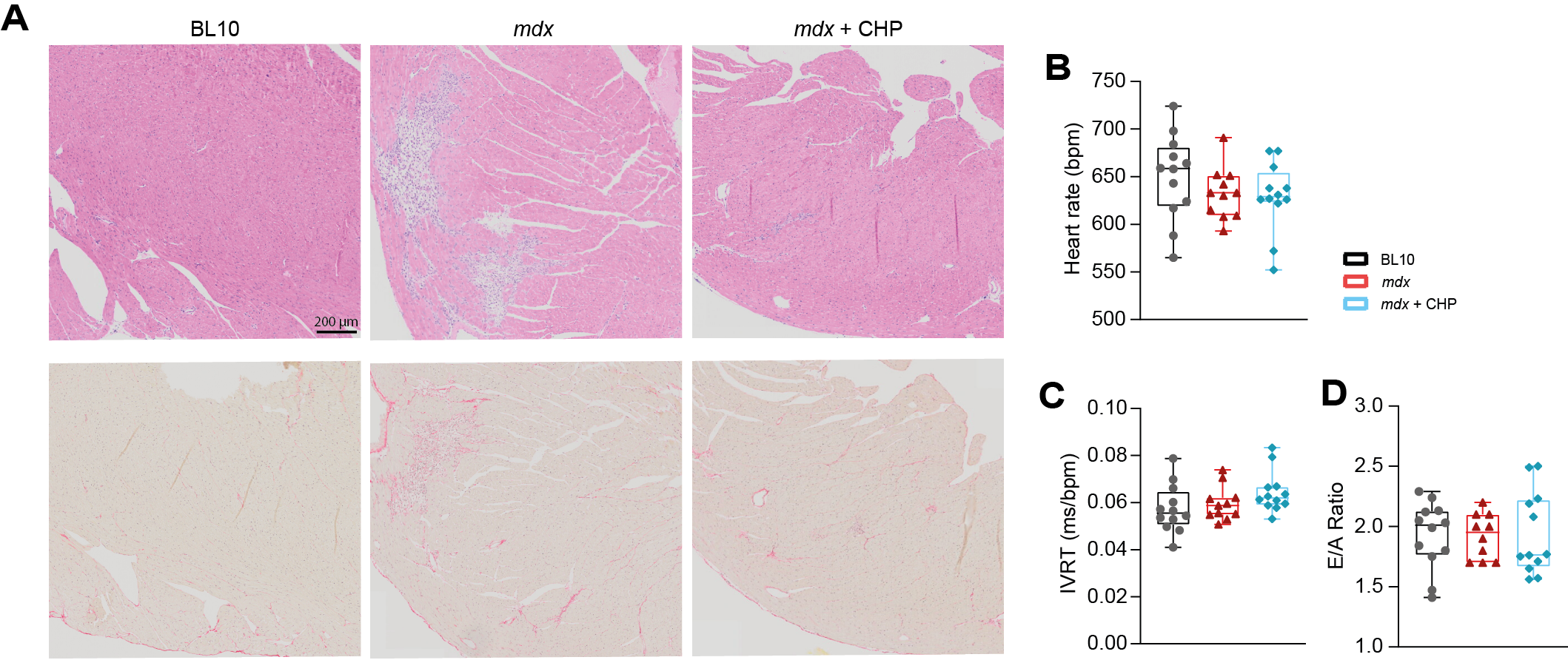


**Figure S2. Histological analysis of heart samples from *mdx* mice.** (A) Representative images of heart samples from BL10, *mdx* and *mdx* mice treated with CHP, stained with hematoxylin and eosin (first line) or with Sirius red for fibrosis (second line). (B) Heart rate. n=12 for BL10, 11 for mdx, 12 for mdx+CHP. (C, D) Echocardiography was performed on BL10, *mdx* and *mdx* mice treated with CHP, at 20 weeks of age. n=12 for BL10, 11 (10 in panel D) for mdx, 12 for mdx+CHP. (C) Isovolumic relaxation time (IVRT) normalized on heart rate. (D) Ratio of the early [E] to late [A] ventricular filling velocities. Whiskers in boxplots represent the min to max range.


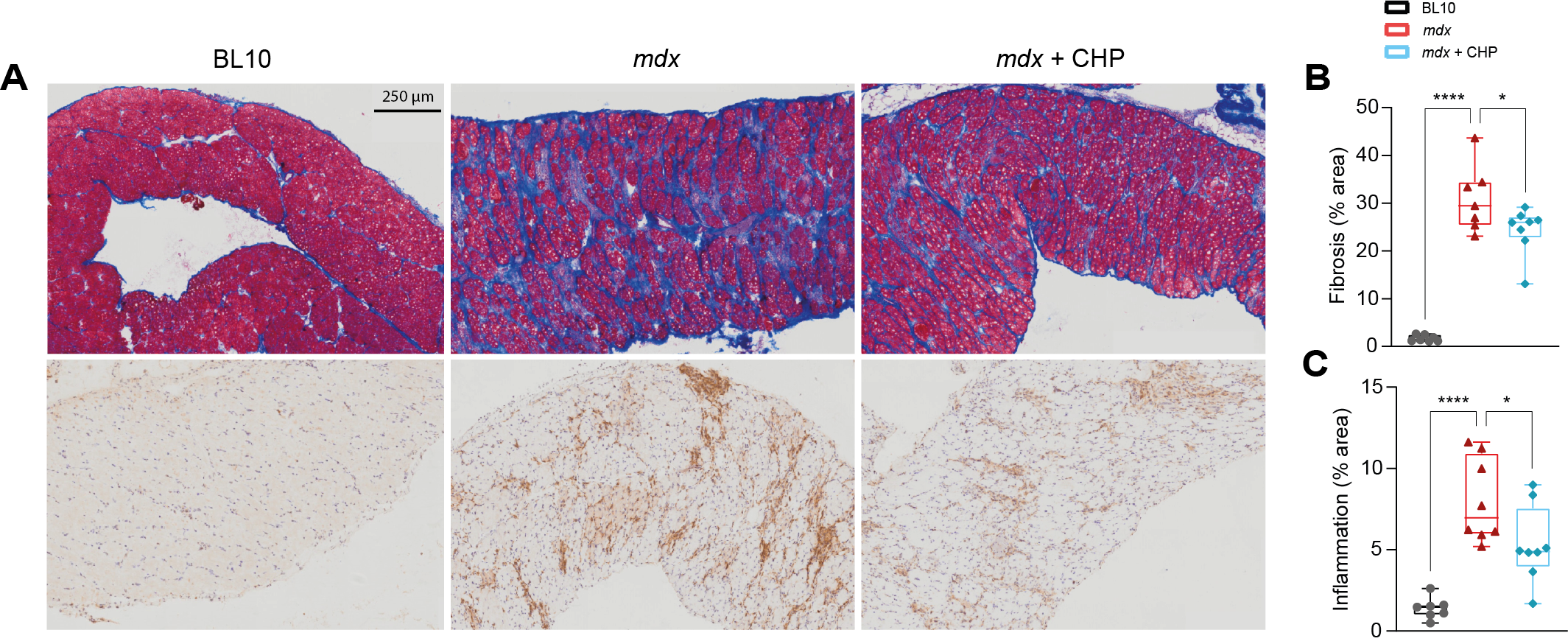


**Figure S3. Histological analysis of diaphragms shows the development of extensive fibrosis and inflammation in mdx mice.** (A) Representative images of diaphragm samples from BL10, *mdx* and *mdx* mice treated with CHP, stained with Masson’s trichrome for fibrosis (first line) or immunostained for CD45 for inflammation (second line). n=7 for BL10, 7 for mdx, 8 for mdx+CHP. (B) Quantification of Masson’s trichrome blue staining in histological images. (C) Quantification of CD45+ staining in histological images. Whiskers in boxplots represent the min to max range. n=7 for BL10, 8 for mdx, 8 for mdx+CHP. (B, C). One-way ANOVA, followed by Dunnett’s multiple comparison test versus *mdx* group, was used for statistical analysis. P values are indicated as follows: * *P*<0.05; **** *P*<0.0001.


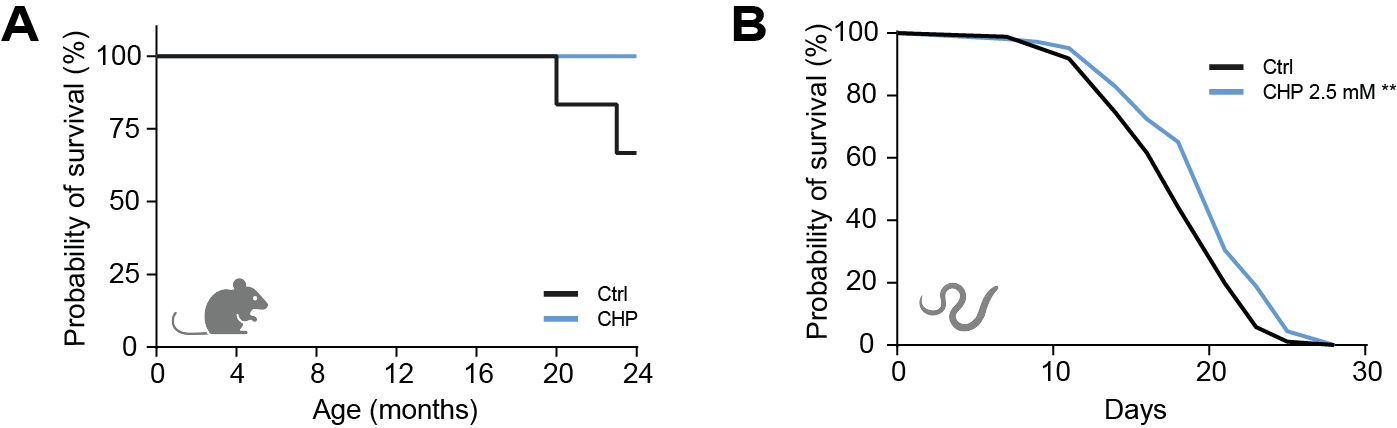


**Figure S4. CHP extended lifespan.** (A) Survival curve of mice from the study outlined in Fig. 5A. n=7. (B) Survival probability of wild-type C.elegans maintained on agar plates containing vehicle or CHP 2.5 mM. Kaplan-Meier survival analysis with Mantel-Cox test was used for the statistical analysis. n>85. ** *P*<0.01.

# **Supplementary tables**

**Table S1.** Body weight and composition of mice in the preventive study outlined in Fig.1A.

| **Mouse group** | **Body weight (g)** | | **Lean mass (%)** | | **Fat mass (%)** | |
| --- | --- | --- | --- | --- | --- | --- |
|  | **week 3** | **week 14** | **week 3** | **week 14** | **week 3** | **week 14** |
| BL10_1 | 18,1 | 30,42 | 81,017 | 68,207 | 9,69 | 16,998 |
| BL10_2 | 16,4 | 27,28 | 80,333 | 71,807 | 11,147 | 10,999 |
| BL10_3 | 16,15 | 30,24 | 81,053 | 72,223 | 9,593 | 11,143 |
| BL10_4 | 19,13 | 28,79 | 80,957 | 73,719 | 9,225 | 10,084 |
| BL10_5 | 21,55 | 29,74 | 78,946 | 66,22 | 11,154 | 17,128 |
| BL10_6 | 19,9 | 27,36 | 80,803 | 69,133 | 9,507 | 15,568 |
| BL10_7 | 22,1 | 30,22 | 79,382 | 66,3 | 9,821 | 18,886 |
| BL10_8 | 14,05 | 29,24 | 80,279 | 75,029 | 10,889 | 9,957 |
| BL10_9 | 16,36 | 32,16 | 80,652 | 67,895 | 9,452 | 17,227 |
| BL10_10 | 15,03 | 30,61 | 78,249 | 69,93 | 11,675 | 15,573 |
| mdx_1 | 14,66 | 33,05 | 81,323 | 80,81 | 9,602 | 6,748 |
| mdx_2 | 11,9 | 33,79 | 81,912 | 81,731 | 9,44 | 5,201 |
| mdx_3 | 11,6 | 28,95 | 80,572 | 80,468 | 11,668 | 5,895 |
| mdx_4 | 12,56 | 32,19 | 80,753 | 78,537 | 10,017 | 8,499 |
| mdx_5 | 10,98 | 30,4 | 82,808 | 81,081 | 8,299 | 5,656 |
| mdx_6 | 21,5 | 30,5 | 81,101 | 77,077 | 9,266 | 10,451 |
| mdx_7 | 20,82 | 30,67 | 81,039 | 82,646 | 8,101 | 9,981 |
| mdx_8 | 21,86 | 29,32 | 80,756 | 78,153 | 8,428 | 9,122 |
| mdx_9 | 9,9 | 34,3 | 83,965 | 79,815 | 8,593 | 7,172 |
| mdx_10 | 10,67 | 34,22 | 82,44 | 79,902 | 9,695 | 6,963 |
| mdx_11 | 11,65 | 35,63 | 80,124 | 81,595 | 11,903 | 6,327 |
| mdx_12 | 11,01 | 33,68 | 83,007 | 80,133 | 8,043 | 5,897 |
| mdx+CHP_1 | 15,15 | 32,6 | 78,036 | 78,912 | 11,348 | 5,506 |
| mdx+CHP_2 | 12,76 | 32,6 | 79,029 | 83,028 | 11,66 | 3,116 |
| mdx+CHP_3 | 13,41 | 33,29 | 79,422 | 79,359 | 9,77 | 8,168 |
| mdx+CHP_4 | 11,55 | 31,32 | 84,205 | 82,459 | 6,916 | 4,828 |
| mdx+CHP_5 | 13,1 | 37,89 | 74,94 | 78,756 | 14,888 | 9,307 |
| mdx+CHP_6 | 16,59 | 36,03 | 77,167 | 80,067 | 14,738 | 7,451 |
| mdx+CHP_7 | 10,8 | 34,27 | 78,483 | 79,42 | 13,15 | 7,742 |
| mdx+CHP_8 | 11,98 | 35,09 | 75,53 | 78,63 | 13,209 | 8,184 |
| mdx+CHP_9 | 13,43 | 33,47 | 77,108 | 77,752 | 14,069 | 9,318 |
| mdx+CHP_10 | 17,57 | 36,93 | 75,44 | 78,307 | 16,385 | 9,372 |

**Table S2.** Body weight and composition of mice in the therapeutic study outlined in Fig.1F.

| **Mouse group** | **Body weight (g)** | | | **Lean mass (%)** | | | **Fat mass (%)** | | |
| --- | --- | --- | --- | --- | --- | --- | --- | --- | --- |
|  | **week 7** | **week 14** | **week 21** | **week 7** | **week 14** | **week 21** | **week 7** | **week 14** | **week 21** |
| BL10_1 | 24,25 | 31,01 | 33,09 | 77,791 | 68,338 | 62,081 | 7,019 | 15,556 | 21,608 |
| BL10_2 | 24,69 | 29,8 | 30,57 | 72,96 | 66,986 | 66,49 | 10,441 | 16,663 | 17,483 |
| BL10_3 | 25,75 | 24,92 | 35,19 | 76,461 | 63,846 | 62,056 | 8,2 | 19,538 | 22,337 |
| BL10_4 | 25,9 | 31,66 | 33,38 | 73,907 | 66,287 | 64,051 | 9,51 | 17,018 | 19,623 |
| BL10_5 | 25,62 | 32,06 | 34,014 | 75,43 | 70,435 | 67,103 | 10,29 | 13,26 | 16,588 |
| BL10_6 | 28,28 | 34,06 | 36,68 | 75,44 | 69,749 | 63,884 | 8,832 | 13,554 | 20,639 |
| BL10_7 | 22,77 | 28,35 | 28,68 | 80,087 | 76,125 | 74,024 | 5,787 | 7,841 | 9,931 |
| BL10_8 | 25,03 | 32,5 | 34,32 | 78,451 | 67,284 | 64,626 | 5,856 | 15,728 | 19,13 |
| BL10_9 | 26,38 | 33,04 | 35,32 | 76,239 | 65,712 | 61,234 | 10,318 | 19,507 | 24,492 |
| BL10_10 | 25,86 | 28,92 | 31,64 | 74,137 | 72,369 | 65,365 | 11,367 | 11,473 | 19,449 |
| BL10_11 | 24,3 | 29,82 | 31,9 | 77,387 | 67,291 | 67,526 | 7,736 | 15,147 | 15,35 |
| BL10_12 | 22,66 | 25,94 | 27,56 | 72,013 | 74,102 | 65,67 | 11,857 | 10,1 | 18,323 |
| mdx_1 | 24,75 | 33,7 | 35,04 | 85,093 | 82,82 | 82,181 | 2,916 | 3,813 | 4,693 |
| mdx_2 | 25,64 | 33,93 | 35,26 | 82,748 | 81,535 | 79,49 | 3,92 | 4,873 | 7,579 |
| mdx_3 | 24,9 | 33,59 | 35,23 | 84,156 | 82,621 | 81,716 | 3,887 | 3,62 | 5,501 |
| mdx_4 | 26,83 | 35,39 | 37,03 | 83,788 | 81,186 | 78,886 | 3,64 | 4,805 | 8,449 |
| mdx_5 | 25,42 | 28,35 | 30,86 | 81,631 | 82,068 | 81,077 | 6,192 | 4,291 | 5 |
| mdx_6 | 25,18 | 29,41 | 31,81 | 82,243 | 83,217 | 81,915 | 5,192 | 3,654 | 5,218 |
| mdx_7 | 25,36 | 30,19 | 31,16 | 84,126 | 78,784 | 78,249 | 5,307 | 7,454 | 7,702 |
| mdx_8 | 25,84 | 32,56 | 36,15 | 81,801 | 82,799 | 81,29 | 6,544 | 3,899 | 6,136 |
| mdx_9 | 27,26 | 30,56 | 33,35 | 80,542 | 81,044 | 81,138 | 5,905 | 4,611 | 6,367 |
| mdx_10 | 28,54 | 33,59 | 36,08 | 80,293 | 79,463 | 77,086 | 8,078 | 6,829 | 9,623 |
| mdx_11 | 28,04 | 34,9 | 37,74 | 81,69 | 79,987 | 77,733 | 6,18 | 6,982 | 9,236 |
| mdx+CHP_1 | 26,5 | 34,67 | 36,56 | 83,355 | 83,044 | 82,712 | 3,981 | 3,341 | 3,633 |
| mdx+CHP_2 | 25,35 | 33,49 | 35,28 | 85,038 | 83,065 | 81,042 | 2,954 | 3,435 | 6,053 |
| mdx+CHP_3 | 27,24 | 33,59 | 35,87 | 84,07 | 80,264 | 81,585 | 4,338 | 5,519 | 5,637 |
| mdx+CHP_4 | 25,7 | 33,49 | 35,92 | 83,162 | 78,677 | 80,294 | 4,754 | 4,626 | 6,794 |
| mdx+CHP_5 | 23,27 | 31,7 | 32,09 | 84,283 | 80,066 | 78,942 | 4,476 | 6,26 | 7,923 |
| mdx+CHP_6 | 25,81 | 31,63 | 32,47 | 84,037 | 78,734 | 76,234 | 3,761 | 8,227 | 10,83 |
| mdx+CHP_7 | 26,35 | 32,66 | 33,65 | 82,582 | 80,276 | 79,403 | 5,876 | 5,809 | 7,434 |
| mdx+CHP_8 | 25,7 | 29,88 | 31,88 | 83,531 | 78,98 | 77,679 | 4,603 | 6,118 | 8,934 |
| mdx+CHP_9 | 26,08 | 29,21 | 31,2 | 81,023 | 81,051 | 78,49 | 6,86 | 5,339 | 7,833 |
| mdx+CHP_10 | 24,2 | 30,65 | 31,7 | 84,158 | 82,44 | 81,212 | 3,699 | 3,504 | 5,382 |
| mdx+CHP_11 | 24,46 | 30,06 | 31,06 | 83,438 | 81,394 | 79,751 | 4,594 | 4,156 | 7,089 |
| mdx+CHP_12 | 22,78 | 29,31 | 31,8 | 81,523 | 81,804 | 81,402 | 5,71 | 4,288 | 5,489 |

**Table S3**. Primer sets for Real-time PCR

|  | Forward (5'-3') | Reverse (5'-3') |
| --- | --- | --- |
| *Myostatin* | ACCCGTCAAGACTCCTACAA | CCTGGGCTCATGTCAAGTTT |
| *Atrogin-1* | TCAAAGGCCTCACGATCACC | TCAAACGCTTGCGAATCTGC |
| *Foxo-1* | CCTTTCCTCCTCCCTCTG | TGCCTCTACTGAATGATTACA |
| *Drp-1* | AGGAGAAGAGGAAGCAAGCG | TAGGCTTTCCAGCACTGAGC |
| *Err-α* | CAGGAGGCAGACACTGAT | CGGATTAAGCAGCAGCAA |
| *Sirt-1* | GTTGACCGATGGACTCCTCAC | GAGCTGGCGTGTGACGTTC |
| *Nrf-2* | CAGCATAGAGCAGGACATGGAG | GAACAGCGGTAGTATCAGCCAG |
| *β-actin* | GGGAAGGTGACAGCATTG | ATGAAGTATTAAGGCGGAAGATT |
